# Individual participant data (IPD) meta-analysis of psychological relapse prevention interventions versus control for patients in remission from depression: a protocol

**DOI:** 10.1136/bmjopen-2019-034158

**Published:** 2020-02-13

**Authors:** Josefien J F Breedvelt, Fiona C Warren, Marlies E Brouwer, Eirini Karyotaki, Willem Kuyken, Pim Cuijpers, Patricia van Oppen, Simon Gilbody, Claudi L H Bockting

**Affiliations:** 1 Department of Psychiatry and Amsterdam Public Health research institute, Amsterdam University Medical Centre - Location AMC, Amsterdam, The Netherlands; 2 Institute of Health Research, College of Medicine & Health, University of Exeter, Exeter, UK; 3 Department of Clinical, Neuro and Developmental Psychology, Amsterdam Public Health research institute, Vrije Universiteit Amsterdam, Amsterdam, The Netherlands; 4 Department of Global Health and Social Medicine, Harvard Medical School, Boston, Massachusetts, USA; 5 Department of Psychiatry, University of Oxford, Oxford, Oxfordshire, UK; 6 Department of Psychiatry, Amsterdam Public Health research institute, Amsterdam University Medical Centre, location VUmc and GGZ InGeest, Amsterdam, Netherlands; 7 Mental Health and Addictions Research Group - Department of Health Sciences, The University of York, York, UK

**Keywords:** depression & mood disorders, individual participant data, psychotherapy, relapse prevention, personalized medicine

## Abstract

**Introduction:**

Psychological interventions and antidepressant medication can be effective interventions to prevent depressive relapse for patients currently in remission of depression. Less is known about overall factors that predict or moderate treatment response for patients receiving a psychological intervention for recurrent depression. This is a protocol for an individual participant data (IPD) meta-analysis which aims to assess predictors and moderators of relapse or recurrence for patients currently in remission from depression.

**Methods and analysis:**

Searches of PubMed, PsycINFO, Embase and Cochrane Central Register of Controlled Trials were completed on 13 October 2019. Study extractions and risk of bias assessments have been completed. Study authors will be asked to contribute IPD. Standard aggregate meta-analysis and IPD analysis will be conducted, and the outcomes will be compared with assess whether results differ between studies supplying data and those that did not. IPD files of individual data will be merged and variables homogenised where possible for consistency. IPD will be analysed via Cox regression and one and two-stage analyses will be conducted.

**Ethics and dissemination:**

The results will be published in peer review journals and shared in a policy briefing as well as accessible formats and shared with a range of stakeholders. The results will inform patients and clinicians and researchers about our current understanding of more personalised ways to prevent a depressive relapse. No local ethics approval was necessary following consultation with the legal department. Guidance on patient data storage and management will be adhered to.

**PROSPERO registration number:**

CRD42019127844.

**Strengths and limitations of this study**

- An individual participant data (IPD) analysis can be a superior method to standard meta-analyses, as it allows us to increase power to detect potential predictors and moderators of treatment.
- This is the first IPD meta-analysis which sets out to evaluate a wide range of psychological relapse prevention interventions for depression.
- This, to our knowledge, is the largest IPD meta-analysis thus far to assess the effects of moderators on relapse and recurrence of depression.
- IPD meta-analyses are limited by the data available, which may limit the number of studies included and subsequently reduce statistical power and limit generalisability.
- Moreover, there may be inconsistencies in terms of how covariates are reported, limiting the number and range of moderators that can be included in the analysis.

## Introduction

Depression is a highly debilitating mental health problem, and one of the leading causes of disability worldwide.^1^ Depression or major depressive disorder (MDD) has a chronic course, and relapse rates are high.^2 3^ Approximately 40%–60% of patients who develop a major depressive episode relapse, and this risk increases up to 90% after three episodes.^4–7^

Recurrence and relapse are terms frequently used synonymously to describe the reoccurrence of depressive symptoms.^8 9^ Both terms indicate a worsening of symptoms and onset of a new episode of depression after a period of no or subthreshold symptomatology. However, they do so at different time points after the initial episode.^8 9^ The clinical and scientific distinctions on these time cut-offs between relapse and recurrence were found to be unhelpful.^10^ Therefore we will use relapse to describe both recurrence and relapse in this manuscript.

Two types of interventions are recommended for relapse prevention for remitted or recovered MDD patients; either continuation of antidepressant medication (ADM) or psychological interventions.^11 12^ ADM or psychological interventions can be offered as a continuation following the therapy on which remission was achieved (continuation therapy), or as sequential interventions, where a different therapy is offered after (spontaneous) remission is achieved.^4 13 14^ Mindfulness-based cognitive therapy (MBCT), preventive cognitive therapy (PCT) and wellbeing therapy (WBT) are examples of psychological relapse prevention interventions used in sequential therapy.^2 4 13 15–19^

A substantial proportion of patients still relapse even after having received a psychological intervention, ADM or a combination of these after remission to prevent relapse.^19 20^ It might be that some psychological intervention types might be more effective, or they have varying levels of effectiveness for different patient characteristics. These characteristics can be separated into prognostic indicators and prescriptive factors.^21^ Prognostic factors affect the course of depression regardless of the treatment provided.^21^ Prescriptive factors, also called ‘moderators’, indicate differential treatment effects across patient characteristics and can be used inform decisions as to which treatment may be best suited to someone with specific characteristics, such as continuation or sequential treatment and/or indicated treatment approaches.^21 22^

A recent meta-analytic review of reviews found that, in recurrent depression, childhood maltreatment, previous depressive episodes, residual symptoms after treatment, co-occurring anxiety disorders as well as rumination were among the strongest prognostic factors in recurrent depression.^23^ In terms of moderators, there was less evidence to suggest any variable could affect treatment selection. Some evidence suggests that residual symptoms at baseline may be potentially be reduced more by cognitive behavioural therapy (CBT) compared with ADM and patients with a history of severe childhood maltreatment may respond better to MBCT compared with treatment as usual (TAU).^23^ However, it remains unclear whether treatment outcomes can be improved if certain interventions are offered to patients with these characteristics.^4 23 24^

An individual participant data (IPD) meta-analysis can provide a unique insight into treatment effectiveness and the effect of individual-level moderators on treatment outcome.^25 26^ IPD analyses are relatively new in the field of clinical psychology and psychiatry.^27 28^ They can provide a more accurate estimate of the true treatment effect and help identify which interventions work better or worse in specific subgroups of patients.^26^ The use of IPD from multiple studies combined can increase the power to detect which patients respond best to treatment within and across studies, something which cannot be assessed with aggregate trial information alone.^29^ Besides, an IPD meta-analysis can adjust for study-level confounding factors which may bias the result of a traditional aggregate meta-analysis.^30^

To our knowledge, only one IPD meta-analysis evaluating psychological interventions versus control for recurrent depression has been conducted thus far.^31^ In this IPD meta-analysis, Kuyken and colleagues estimated the effectiveness of MBCT versus control (active or non-active) and aimed to establish the effect of moderators on treatment outcome. Among nine included studies with 1258 participants, the authors performed a time to event (depressive relapse) IPD meta-analysis which produced an HR of 0.69 (95% CI 0.58 to 0.82), indicating a reduced risk of relapse in those participants who received MBCT compared with participants in the control group. No differential treatment effects across participant characteristics were found. In this review, depression baseline scores (indicative of incomplete recovery) predicted a stronger effect at follow-up. Thus, patients with higher symptomatology at the start of treatment seemed to benefit most. Kuyken *et al* 31 only included MBCT, a range of other forms of psychological interventions were not included (ie, CBT, PCT or interpersonal therapy). Including these would increase power to assess moderators of treatment, establish effects of different psychological interventions versus control, as well as allow for an evaluation of the interaction between therapy type and moderator.

This study aims to answer three research questions which remain pertinent from the literature, namely:

1. What are the effects of different preventive psychological interventions on reducing the risk of relapse versus control?
2. What are the predictor variables associated with an increased risk of relapse for patients receiving a psychological intervention?
3. What factors (moderators) may predict which participant responds best to which type of intervention?

## Methods

### General approach

This IPD meta-analysis is registered on PROSPERO and any key changes or amendments will be documented there. The protocol is registered retrospectively as searches and aggregate study-level extractions were already conducted in 2018, and the last search was conducted on 13 October 2019. The Preferred Reporting Items for Systematic Reviews and Meta-Analyses IPD statement will be followed for the reporting of this study.^32^

### Systematic review to identify eligible papers

#### Eligibility criteria

##### Types of studies

Eligible studies must have a randomised controlled study (RCT) design and be written in the English language.

##### Types of interventions

Included studies will examine the effects of psychological relapse prevention interventions, and we used the following definition: ‘a modality of treatment in which the therapist and patient(s) work together to ameliorate psychopathological conditions and functional impairment through focus on the therapeutic relationship; the patient’s attitudes, thoughts, affect and behaviour; and social context and development’.^33^ The intervention can be delivered in any modality or setting, such as face to face, in a group format or online. Examples include MBCT, PCT, continuation cognitive therapy, or WBT.^13 14 17 19 34^ Sequential treatment combinations, that is, studies where a different relapse prevention therapy starts directly after treatment for acute depression,^13 14^ will be included as long as patients were randomised to a relapse prevention intervention after reaching remission or recovery (as defined by the study authors) from the acute episode. As we are interested in the overall effect of psychological interventions versus control, studies where participants were randomised to taper from ADM as they receive the psychological intervention will be excluded.

##### Types of comparators

To answer our first question (estimate of treatment effect), at least one non-psychotherapeutic control group should be available. Eligible control conditions include TAU, ADM and active psychological control group. For our second question (prognostic indicators) for patients receiving psychological interventions, any control group can be included. For our third question (moderator analysis), which assesses what works for whom, at least one non-psychological control group should be available.

##### Types of participants

Participants aged 18 and over were included. Studies, where participants were only included if they had an onset after the age of 65, were excluded. As also specified by Brouwer (2019), the factors causing and contributing to the first onset of depression in the 65 and above age range may differ from lower age groups.^35–37^ Participants in trials need to have had at least one prior diagnosis of MDD established by an independent clinical interview and/or healthcare provider who was not involved in the study. The randomisation and subsequent treatment or TAU should have been during the remission, response or recovery phase. Participants were required to be in remission from at least one episode of MDD. Remission is classified as a period of at least 8 weeks, where participants had no or subthreshold clinical symptoms.^4 38^ Sequential treatment combinations were included as long as participants in the intervention group achieved remission or response according to the authors of the study. Studies were excluded when participants were in treatment for another mental disorder as classified by the Diagnostic and Statistical Manual of Mental Disorders (DSM)-V, DSM-IV, or DSM-III criteria.^39 40^ ^41^

#### Outcomes

##### Primary outcome

The primary outcome of the study will be time to depressive relapse at any point of follow-up measured in weeks. Depressive relapse needs to be determined via an independent examiner/assessor via a diagnostic interview, for example, the Structured Clinical Interview for DSM-IV Axis 1 Disorders,^42^ the Mini-International Neuropsychiatric Interview,^43 44^ the Composite International Diagnostic Interview^45^ or the Hamilton Depression Rating Scale.^46^

##### Covariates

We are interested in patient characteristics which predict relapse of depression regardless of treatment allocation (predictor variables), and those that may affect outcome based on treatment allocation (moderators). Sometimes these terms are used interchangeably. However, in this manuscript we use these terms to differentiate between overall treatment outcome predictors and factors moderating treatment response (ie, who responds better to one treatment vs a control condition or vice versa). Patient characteristics will be included in the analyses if they are consistently reported and available across datasets and justify inclusion based on prior literature that identifies them as potential predictors or moderators.^4 23 47^

We will ask the individual studies to provide data on the following set of variables which could either be predictors or moderators of effect.

Age at baseline, gender, ethnicity, country of birth, education status, employment status, marital status, number of previous depressive episodes, age of onset of first episode of depression, time spent in remission since last episode, duration of past episode of depression, stable or unstable remission since last episode, history of childhood trauma, previously received psychotherapy for MDD (including type and time since last session), previously received medication for MDD (including type, dosage and time since last intake), comorbid anxiety disorder, comorbid mental health disorder, comorbid physical health disorder, antidepressant exposure at baseline, baseline depression symptoms, baseline anxiety symptoms, baseline quality of life symptoms.

##### Timing of outcome measures

The primary outcome, time to depressive relapse, will be included for all participants, regardless of variation in follow-up duration across studies. We will consider censoring follow-up time, for example at 60 weeks, contingent on the length of follow-up across studies.

##### Secondary outcomes

Secondary outcomes include depressive symptomatology at follow-up, anxiety symptoms and quality of life. These will be collected and may be used for future analysis.

### Searches for study identification and selection

PubMed, PsychINFO, Embase and Cochrane Central Register of Controlled Trials were searched. Index and free terms, jointly with Boolean operators, were used on four tiers, namely: (1) depressive disorder, (2) recurrence and relapse, (3) preventative interventions, (4) RCT. Online supplementary appendix 1 shows the search terms for PubMed. References from previous meta-analyses were screened to ensure no RCTs are missing.^14 19 48–51^ Key authors in the area of relapse prevention for depression were consulted for additional literature and unpublished manuscripts.

10.1136/bmjopen-2019-034158.supp1 Supplementary data

The last searches for this IPD were completed on 13 October 2019, three researchers independently screened search results (JB screening all and a research assistant or collaborator conducting the second screen). Full-text screens were conducted by two independent researchers. Outcome data for all studies are being extracted independently by at least two independent researchers and are then being merged and checked by JB. Discrepancies were discussed with the researchers involved in extraction and resolved via discussion with MB.

### Quality assessment

Study quality will be assessed by two reviewers who will independently evaluate the studies based on six criteria for risk of bias from the Risk of Bias tool by the Cochrane Collaboration.^52 53^ The following criteria will be assessed: (1) random sequence generation; (2) allocation concealment; (3) blinding of outcome assessors; (4) incomplete outcome data; (5) selective outcome reporting; (6) other threats to validity (similar groups, cointerventions, compliance and similar timing of outcome assessment). Studies will be rated on each criterion with either ‘low risk’ ‘high risk’ ‘unclear risk’. A minimum of five criteria with a ‘low risk’ rating qualified as the overall low risk of bias. Only data that are published in the full-text paper of the trial will be evaluated and assessed by two independent assessors, to avoid potential imbalance between studies that can share data and those that are not able to do so.

### Patient and public involvement

During the development of this protocol, no direct patient involvement has taken place. However, we are planning to consult with people with lived experience during the interpretation and dissemination of the study results. Results will be disseminated in peer review journals but also a coproduced evidence briefing in collaboration with the Mental Health Foundation. We have set up a consortium of all collaborating authors on this IPD (and other researchers as well as other stakeholders interested in relapse prevention of depression) titled "International Taskforce For RelApse prevention of depression (ITFRA)". This taskforce aims to help disseminate the research and raise awareness with the public and clinical community.

### IPD data collection and aggregation

#### Invitation of authors

All corresponding authors of the selected articles for inclusion will be contacted via email by the senior authors of this article with an invitation to participate in the study. The letter includes details regarding the study group, the study proposal, the goals of the analysis, the variables of interest and includes a request to share raw data from the trial participants for this study. When there is no response from the author within 4 weeks, we will try a second time. If this fails, a second senior author will be contacted. We will continue until we have reached at least three authors. If none of the authors responds or if all the authors indicate that the data are unavailable or that they are unable to share due to access restrictions, it will be noted that the study data were unavailable. We will send a maximum of four reminders until we exclude the study as being unavailable.

#### Data checking and integrity

After accepting the invitation to collaborate and signing the data transfer agreement, the authors will be asked to share their data via a secure data transfer portal available to the Amsterdam University Medical Centre—Location AMC. The received data will be reviewed to assess the completeness and accuracy of the dataset. If any inconsistencies are present (missing data, inconsistencies or extreme values, discrepancies between the trial report and the data), the issue will be discussed with the study authors who will be contacted for clarification. The study progress and discrepancies will be recorded.

#### Creating a database and aggregation

A template spreadsheet with study characteristics and outcome data will be created. Once data have been checked and standardised, it will be merged into the final file for analysis. All individual datasets will be merged into one large IPD dataset. Once all data have been merged into a final dataset, it will be rechecked for accuracy by a researcher in the study team, by comparing participant numbers, descriptive data and relapse/recurrence data to the reported data in the peer-review article.

### Statistical analysis

To determine the final selection of potential predictors and moderators, we will identify reported covariates across studies and identify ways that covariates can be appropriately standardised across studies, for example, by collapsing categories. Baseline covariates will be included in the modelling if the covariate has at least 40% available data (ie, non-missing) in at least three studies.^54^

To examine the overall effects of the interventions compared with control (research question 1) and assess the modifying effect of study and individual-level variables on prognostic and predictive value (research questions 2 and 3), we will conduct one-stage and two-stage random-effects IPD meta-analyses using Stata v.14.^55^

Our primary meta-analysis method will be the one-stage random-effects approach; we will seek to perform one-stage random-effects meta-analyses for the time to event outcomes using hierarchical flexible parametric models.^56^ A random-effects meta-analysis was chosen because we are aware of clinical heterogeneity in the included trials (eg, due to differences in study populations, types of psychotherapy, or differences in the control group), which may result in statistical heterogeneity. Should such models fail to converge, we will then perform Cox proportional hazards models stratified by study (one-stage fixed effect approach).

A two-stage method for time to event data calculates the HR for relapse for each study individually, using a Cox proportional hazards model; these HRs will then be combined in the IPD meta-analysis. If only rate ratio data are available, we aim to calculate an estimate of HR data from rate ratio data by the logarithms of event-free proportions.^57^ We will use the DerSimonian and Laird^58^ random effects method to combine the results of the individual studies and will apply the Hartung Knapp-Sidik-Jonkman method correction to account for uncertainty in τ^2^.^59 60^

We aim to answer our first research question (estimate of effect) via an IPD meta-analysis on a maximum of four pairwise comparisons: (1) psychotherapy versus ADM, (2) psychotherapy versus TAU, (3) psychotherapy versus active control (ie, placebo or active psychological intervention), (4) psychotherapy versus any non-psychotherapy control. All analyses will use the intention to treat approach, whereby all participants will be included in the analyses according to their randomised allocation irrespective of treatment received. Primary analyses will use clinical and demographic participant baseline characteristics and time to event outcome data.

To assess statistical heterogeneity, we will assess I^2^ 26 derived from two-stage meta-analysis models, with 0% indicating no heterogeneity, 25% low heterogeneity, 50% moderate heterogeneity and 75% as being high heterogeneity.^26^ A 95% prediction interval will be calculated to evaluate the potential range of the treatment effect when applied in an individual study setting.^28^

#### Missing data

The percentage of individual participant missing data (baseline characteristics, event status and time to event or censoring) will be recorded for each study. We plan to use multiple imputation on participant baseline characteristics which are missing individually within studies reporting the specified characteristic^61^ and a sensitivity analysis would be performed using observed and imputed data.

#### Sensitivity analysis

To assess whether various sources of heterogeneity affect the overall effect size and the robustness of the IPD findings, we will conduct sensitivity analyses. Sources of heterogeneity, including study level characteristics such as the risk of bias, year of publication, setting (community, primary or secondary care), duration of follow-up, country of study, will be explored. We will also explore differences between one and two-stage IPD meta-analytic approaches.^62^

#### Aggregate data meta-analysis

We will investigate the possibility of inclusion bias by reporting the characteristics of eligible trials for which data were sought but not obtained. If suitable results are available for these studies, we will perform two-stage meta-analyses to incorporate the results of these trials with those of the trials where IPD was obtained. Potential publication bias will be estimated by funnel plot inspection and by use of Egger’s test for asymmetry.^63^

#### Predictors and moderators

To investigate the effects of baseline participant characteristics on relapse regardless of the psychological intervention offered (research question 2), each of the predictors will be entered into a time-to-event model with the predictor as the independent variable (together with treatment allocation) and time to relapse as the dependent variable. To account for clustering of patients within a study we will include a study as a variable within the model, either as a random or fixed effect. Individual predictor v will be selected based on p values. We will initially include variables that are associated with the outcome with p<0.10, in regression models, including an individual variable and treatment allocation only. These variables will then be combined in a further model; any variables with p>0.05 will then be removed, which follows the criteria by Heffner *et al* 64 as described in Ahmed *et al*.^65^ Each variable that has been removed will then be added individually to the model where all variables have p<0.05 and will be included if their p value is <0.05. In this way, predictors of time to depressive relapse will be identified. We will also assess the model fit by Akaike’s Information Criterion.^66^

To investigate differential treatment effects across different participant subgroups (research question 3), we will perform a series of models adding the interaction term between each moderator and treatment allocation (each model will include only one interaction effect). In the two-stage model, we will first estimate the interaction at study level and will then combine interaction estimates in a random effects-meta-analysis. Potential moderators will be centred within the study to avoid ecological bias; continuous covariates will be centred around the study mean; binary covariates will be centred around the proportion with the characteristic.^28 67^ Treatment allocation for the above model will be categorised by psychological intervention versus non-psychological intervention in the first instance.

## Discussion

This IPD meta-analysis can provide up to date treatment efficacy estimates and has the potential to establish whether there are any moderators and predictors of treatment effect. This could help answer the ‘what works for whom’ question we are hoping to answer. The IPD has several strengths, first it allows us to look at individual-level data rather than study-level data which give us the possibility to assess whether specific baseline and treatment characteristics predict or moderate time to relapse with more specificity and more power. Moreover, we will be able to study our outcome of interest more precisely, using the individual time to event data rather than aggregate information presented at study level.^68^ Various factors may hamper the process and interpretation of an IPD meta-analysis. There may be inconsistencies across studies regarding which variables are reported, and how they are reported, thus limiting our ability to assess the effect of these potential predictors and moderators of treatment. There may be some inclusion bias, as we may not be able to obtain IPD for all eligible studies; however, where possible, sensitivity analyses with the inclusion of published aggregate data will address this issue.

This is to our knowledge the first IPD meta-analysis that attempts to establish the effects of a range of psychological relapse prevention strategies compared with control conditions, as well as assessing whether any predictors or moderators might affect the risk of relapse for patients receiving a psychological intervention or a control intervention.

This study has the potential to inform clinicians, healthcare providers and people who have had a previous episode of depression. Firstly on the relative effectiveness of different approaches for prevention of depressive relapse and secondly this study may also offer further indications on overall risk factors and what may work for whom in preventing relapse for patients who have experienced a previous episode of depression.

### Ethics and dissemination

We will disseminate the work via peer review publications. Day to day oversight and management of the database will be by JB and analyses will be conducted by JB and FCW. The dataset is not open access. Researchers may potentially receive access to the database on request pending on institutional approvals on data transfer and ethics and approval of all the collaborating co-authors for sharing the data beyond the ITFRA consortium. Patient privacy will be ensured by adhering to the Amsterdam University Medical Centre—location AMC guidance on research participant data management and storage. This covers a range of data protection and storage measures including (1) setting up inter-institutional data sharing agreements prior to data sharing, (2) sharing only pseudonymised (de-identified) data, (3) secure data sharing and data storage.
