# Supplementary material for: Individual participant data (IPD) meta-analysis of psychological relapse prevention interventions versus control for patients in remission from depression: a protocol
Source: BMJ Open. 2020 Feb 13;10(2):e034158. doi: 10.1136/bmjopen-2019-034158 (PMC7044815; doi:10.1136/bmjopen-2019-034158)
Supplement: Supplementary data [file bmjopen-2019-034158supp001.pdf]

PubMed:

((((((("depressive disorder"[MeSH Terms] OR "depression"[MeSH Terms] OR depress*[Title/Abstract] OR affective disorder[Title/Abstract] OR affective disorders[Title/Abstract] OR dysphoria[Title/Abstract] OR dysthymia[Title/Abstract] OR depressed mood[Title/Abstract]) OR mood disorder[Title/Abstract]))) AND (("recurrence"[MeSH Terms]) OR ("recur*"[Title/Abstract]) OR ("chroni*"[Title/Abstract]) OR (relaps*[Title/Abstract]) OR ("remi*"[Title/Abstract]) OR ("treatment resistant"[Title/Abstract]))) OR (("recurrent depression"[MeSH Terms]) OR ("treatment resistant depression"[Title/Abstract])))) AND ("secondary prevention"[MeSH Terms] OR "prevention"[MeSH] OR "preventive [MeSH Terms]" OR "maintenance"[Title/Abstract]) OR ("continuation"[Title/Abstract]) OR ("prophyla*"[Title/Abstract]) AND (("randomized controlled trial"[Publication Type] OR controlled clinical trial[Title/Abstract] OR random*[Title/Abstract] OR prevention and control[Title/Abstract]))
